# NtRBP45, a nuclear RNA‐binding protein of *Nicotiana tabacum*, facilitates post‐transcriptional gene silencing

**DOI:** 10.1002/pld3.294

**Published:** 2020-12-24

**Authors:** Wangbin Zhang, Zongcai Zhu, Peixiu Du, Chao Zhang, Hailin Yan, Wenguo Wang, Weimin Li

**Affiliations:** ^1^ College of Plant Science Tarim University Alar PR China; ^2^ Southern Xinjiang Key Laboratory of IPM Tarim University Alar PR China; ^3^ Biotechnology Research Institute Chinese Academy of Agricultural Sciences Beijing PR China; ^4^ Key Laboratory of Development and Application of Rural Renewable Energy Ministry of Agriculture and Rural Affairs Chengdu PR China

**Keywords:** antiviral defense, post‐transcriptional gene silencing, RNA binding protein, siRNA accumulation

## Abstract

The tobacco RBP45 is a nuclear RNA binding protein (RBP). In this study, we identified that the gene expression of NtRBP45 was significantly up‐regulated upon the *Tobacco mosaic virus* infection and the central region of the protein accounted for its nuclear localization. In particular, using a green fluorescent protein‐based transient suppression assay, we uncovered that the transiently overexpressed NtRBP45 was able to enhance local post‐transcriptional gene silencing (PTGS), facilitate siRNA accumulation, and compromise the RNA silencing suppression mediated by *Tomato aspermy virus* 2b protein. Deletion mutagenesis showed that both the N‐ and C‐terminal regions of NtRBP45 were necessary for enhancing PTGS. The data overall indicated a novel RNA silencing factor that might participate in antiviral defense.

## 1 INTRODUCTION

RNA silencing or RNA interference (RNAi) is an evolutionarily conserved eukaryotic mechanism that has a fundamental role in antiviral defense with small interfering RNAs (siRNAs) as a central player (Ding & Voinnet, 2007; Yang & Li, 2018). The siRNAs have 21–24 nucleotides (nt) in size, and are converted from the double‐stranded RNAs (dsRNAs) that originate from transposons (Aravin et al., 2001), transgenes (Wang & Metzlaff, 2005), and viral replicative intermediates (Cogoni & Macino, 2000). In the basic process of antiviral silencing, the dsRNAs are first processed by the type III endoribonuclease DICER or Dicer‐like (DCL) proteins (Liu et al., 2009; Zhang et al., 2004), resulting in siRNA duplexes that are subsequently stabilized via 2′‐O‐methylation at the 3′ terminal nucleotide by a methyltransferase HUA Enhancer 1 (HEN1) (Yang et al., 2006). One strand of the methylated siRNA duplex is in turn loaded onto Argonaute (AGO) protein‐containing RNA‐induced silencing complex (RISC) or RNA‐induced transcriptional silencing complex (RITS), and serves as guide RNA to recognize and target the viral nucleotide sequences via sequence complementarity (Ding & Voinnet, 2007; Sidahmed et al., 2014). RISC specifically causes RNA cleavage or translation inhibition at the post‐transcriptional level and plays a role in host defense against both RNA and DNA viruses, whereas RITS counteracts only DNA viruses by inducing DNA methylation (Csorba et al., 2015).

In addition to DICERs, AGOs, and HEN1 referred to above, a number of proteins are known to be involved in the siRNA‐mediated antiviral silencing. For example, DRB4, a dsRNA‐binding protein, has been defined as an antiviral silencing factor that contributes to siRNA biogenesis by interacting with and regulating the dicing activity of DCL4 (Fukudome et al., 2011; Qu et al., 2008). Moreover, RNA‐dependent RNA polymerases (RDRs), as well as Suppressor of GENE SILENCING (SGS) proteins and SILENCING DEFECTIVE (SDE) proteins, have been shown to be engaged in the secondary siRNA production during the amplification of antiviral RNA response (Csorba et al., 2015; Peragine et al., 2004; Yang & Li, 2018). In this study, we demonstrated that a tobacco RNA‐binding protein (RBP) NtRBP45 was a novel antiviral silencing factor, which could promote siRNA accumulation, enhance RNA silencing at the post‐transcriptional level, and in particular compromise the suppression activity of a viral silencing suppressor *Cucumovirus* 2b.

## 2 MATERIALS AND METHODS

### 2.1 Plant materials and microbial strains

The plants of *Nicotiana tabacum*, *N. benthamiana*, and transgenic *N. benthamiana* homozygous for the GFP transgene (line 16c) (Ruiz et al., 1998) were maintained in a greenhouse at 25°C. *Escherichia coli* DH5α was grown on Luria‐Bertani (LB) medium at 37°C and *Agrobacterium tumefaciens* EHA105 was grown on Yeast Extract Broth (YEB) at 28°C.

### 2.2 Reverse transcription‐quantitative real‐time PCR assay

The fully expanded leaves of 16 *N. tabacum* plants at 6‐week old were mechanically inoculated with *Tobacco mosaic virus* (TMV) strain U1 as described (Dawson et al., 1986). The inoculated leaves were harvested at 24 hr post‐inoculation (hpi), 48 hpi, 72 hpi, and 96 hpi, respectively, and the healthy leaves at the similar development stage were collected as a control. Total RNA of the leaf tissues was extracted using TRIzol Reagent (Invitrogen), followed by reverse transcription (RT) with PrimeScript^TM^ RT reagent Kit with gDNA Eraser (Takara, JP) and oligo(dT)12‐18 to generate the first‐strand cDNA. By TB Green Premix EX Taq II (Tli RNaseH Plus) (Takara, JP) and gene‐specific primers (Table S1) corresponding to *NtRBP45* (GenBank No. XM_016610305), quantitative real‐time PCR (qPCR) was performed on a Step One Plus Real‐time PCR System (Thermo Fisher Scientific), with cycling conditions as follows: pre‐denaturation for 30 s at 95°C, followed by 40 cycles of amplification (95°C for 5 s, 55°C for 30 s, and 72°C for 30 s). The *N. tabacum* actin gene (GenBank No. XM_016658252.1) was employed as an internal reference. The experiment was performed with three biological replicates, each of which consisted of three technical replicates. The relative expression value of *NtRBP45* was calculated by the 2‐^ΔΔ^Ct method and transformed to fold change. Statistical analyses were conducted using the Student's *t* test (SPSS 10.0).

### 2.3 Plasmid constructs

The open reading frame (ORF) of *NtRBP45* that encodes 409 amino acids (aa) was cloned with the primer pair RBP45‐F/RBP45‐R (Table S1) and inserted into pMD18‐T (Takara, JP), resulting in pRBP45 that was used as PCR template in the follow‐up experiments. To generate constructs for investigating the subcellular localization of NtRBP45, the *NtRBP45* gene was amplified with the primer pair RBP45gfp‐F/RBP45gfp‐R (Table S1) and cloned into the *Kpn* I/*Xho* I double‐digested pCAMBIA1300‐35S‐GFP (Liu et al., 2019) to produce pRBP45‐GFP carrying an in‐frame gene fusion between *NtRBP45* and *gfp*. With the same strategy, the nucleotide sequences of four NtRBP45 deletion mutants, including RBP45N^Δ^ corresponding to 66–409 aa of NtRBP45, RBP45C^Δ^ to 1–370 aa, RBP45N^Δ^C^Δ^ to 66–304 aa, and RBP45N to 1–66 aa (Figure S1), were individually amplified with the primers listed in Table S1, and fused with *gfp* to generate pRBP45N^Δ^‐GFP, pRBP45C^Δ^‐GFP, pRBP45N^Δ^C^Δ^‐GFP, and pRBP45N‐GFP. In addition, to create the constructs for exploring the role of NtRBP45 in RNA silencing, the coding sequences of NtRBP45 and its deletion mutants described above were individually amplified with the specific primers (Table S1) and inserted into the *Nco* I/*Bst*E II double‐digested pCAMBIA1305.1, resulting in pCAM‐RBP45, pCAM‐RBP45N^Δ^, pCAM‐RBP45C^Δ^, pCAM‐RBP45N^Δ^C^Δ^, and pCAM‐RBP45N. With the same strategy, pCAM‐TAV2b bearing the *Tomato aspermy virus 2b* gene (*TAV2b*; GenBank No. NC_003838.1) was constructed as a control for evaluating the role of RBP45 in RNA silencing. All the constructs were verified by nucleotide sequencing.

### 2.4 Confocal microscopy

The plasmids pRBP45‐GFP, pRBP45N^Δ^‐GFP, pRBP45C^Δ^‐GFP, pRBP45N^Δ^C^Δ^‐GFP, and pRBP45N‐GFP were individually transformed into the component *A. tumefaciens* EHA105 cells, which, in turn, were infiltrated into the fully expanded leaves of *N. benthamiana* at 6 weeks old as previously described (Van der Hoorn et al., 2000). At 60 hpi, the infiltrated leaves were collected to visualize the GFP fluorescence using an LSM700 confocal microscope (Zeiss) at an excitation wavelength of 488 nm.

### 2.5 GFP imaging and RNA gel blot analysis

The pCAM‐RBP45, pCAM‐RBP45N^Δ^, pCAM‐RBP45C^Δ^, pCAM‐RBP45N^Δ^C^Δ^, and pCAM‐RBP45N were individually introduced into *A. tumefaciens* EHA105. The resulting bacteria were then cultured to an optical density (OD) at 600 nm of 1 and mixed in equal volume with the *Agrobacterium* cells harboring pCAMBIA1300‐35S‐GFP at a similar OD. The resulting mixtures were infiltrated into leaves of the *N. benthamiana* line 16c at 6 weeks old as previously described (Voinnet et al., 2000). The infiltrated leaves were visualized at 3 dpi under UV light with a Black Ray B 100 AP lamp and photographs were taken by a Nikon D7100 digital camera.

Total RNA of the infiltrated leaf tissues (3 dpi) was isolated by TRIzol reagent (Invitrogen). For the gel blot analysis of GFP mRNAs and siRNAs, 15 and 80 µg of total RNA were separated on 1.2% agarose‐formaldehyde gel and 15% denaturing polyacrylamide‐7 M urea gel, respectively, and transferred to Hybond‐N^+^ membrane (Amersham). The membranes were hybridized with the probes specific to the *gfp* sequence, using the digoxigenin (DIG)‐labeled RNA probes complementary to the ORF of GFP in the case of mRNA detection, or the DIG‐labeled DNA probes corresponding to nt 280–319 and nt 429–468 of *gfp* in the case of small RNAs.

## 3 RESULTS

### 3.1 Expression of the tobacco NtRBP45 was elevated upon TMV infection

**FIGURE 1:**
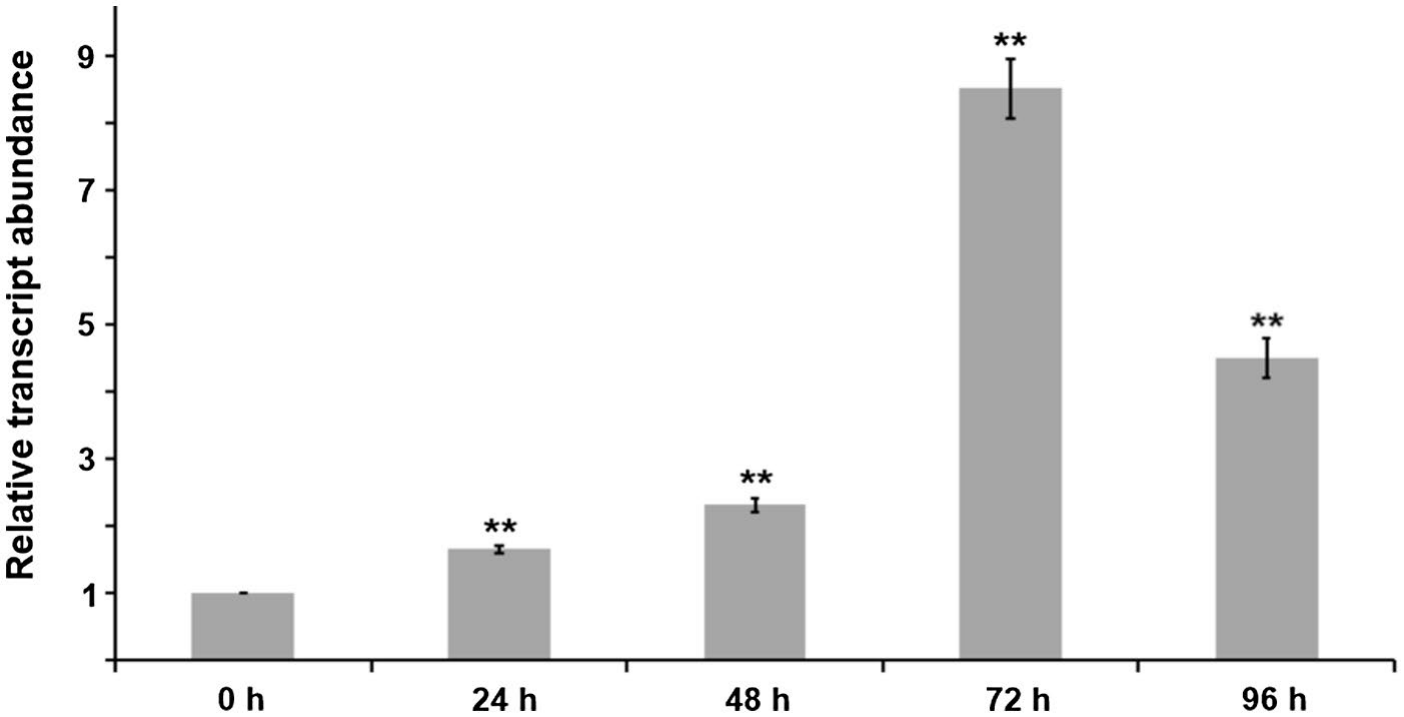
Expression of NtRBP45 in *Nicotiana tabacum* was significantly increased upon *Tobacco mosaic virus* infection. Total RNA was extracted from the healthy tobacco leaves (referred to as 0 hr) as well as the TMV‐infected leaves were individually collected at 24 hpi, 48 hpi, 72 hpi, and 96 hpi, and used to detect the relative expression levels of *NtRBP45* with RT‐qPCR. Three biological replicates (each consisting of three technical replicates) were performed. The *N. tabacum* actin was used as an internal reference, and the *NtRBP45* expression levels at 24 hpi, 48 hpi, 72 hpi, and 96 hpi were individually normalized to that at 0 hr, which was set to 1. Bars represent the mean ± standard error, and asterisks indicate significant differences according to a Student's *t* test (**p < .01)

The plant *RBP45* has a constitutive expression pattern in different organs, such as leaves, roots, and stems (Lorković et al., 2000; Peal et al., 2011). A previous study has shown that the expression levels of two *Arabidopsis RBP45* genes, *AtRBP45a*, and *AtRBP45b* are up‐regulated in response to acute ozone exposure (Peal et al., 2011), implying the importance of RBP45 in abiotic stress response. Here, we examined the expression of *NtRBP45* in the TMV‐infected tobacco leaves (collected at 24 hpi, 48 hpi, 72 hpi, and 96 hpi, respectively), with the healthy leaves (hereafter referred to as 0 hr) as a control. RT‐qPCR analysis disclosed that the transcript level of *NtRBP45* was significantly elevated during the course of the 96‐hr infection and peaked at 72 hpi with an 8.5‐fold increase compared with that at 0 hr (Figure 1). The data showed the induced expression of NtRBP45 upon viral pathogen infection, thus suggesting that the plant RBP45 was also implicated in biotic stress response.

### 3.2 The central region of NtRBP45 contributed to the nuclear localization of the protein

**FIGURE 2:**
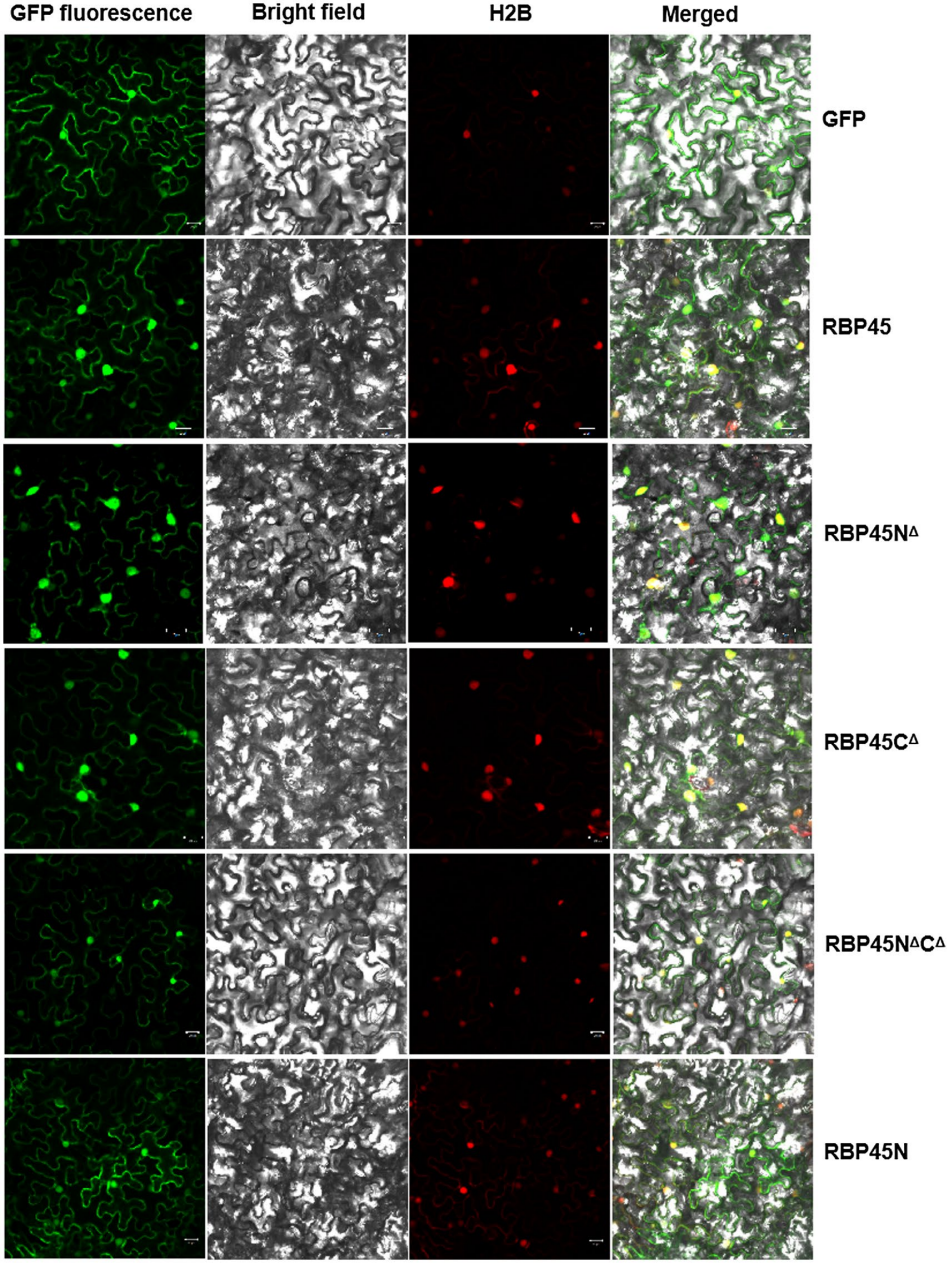
Subcellular localization of GFP fusions with NtRBP45 as well as the deletion mutants RBP45N^Δ^, RBP45C^Δ^, RBP45N^Δ^C^Δ^, and RBP45N. The corresponding constructs were individually delivered into the *Nicotiana benthamiana* leaves via agroinfiltration, and the green fluorescence was viewed by confocal microscopy after 48 hr post‐inoculation. H2B‐RFP was used as a marker for the nucleus. Scale bars represent 20 µm

RBP45 is a nuclear‐localized protein that contains the N‐terminal region enriched in glutamine, the central region with three RNA‐binding domains (RBD), and the C‐terminus with a glycine and tyrosine‐rich domain (Lorković et al., 2000). Unfortunately, the bioinformatic approaches available so far gave ambiguous results on the potential nuclear localization signal (NLS) within NtRBP45 (data not shown). To map the sequence required for nuclear localization, we created four deletion mutants (RBP45N^Δ^, RBP45C^Δ^, RBP45N^Δ^C^Δ^, and RBP45N) according to the putative N‐terminal, central an C‐terminal regions of NtRBP45, and further individually fused with GFP. The resulting fusion proteins were transiently expressed in the *N. benthamiana* leaves and observed with confocal microscopy. As shown in Figure 2, the green fluorescence of RBP45N^Δ^‐GFP, RBP45C^Δ^‐GFP or RBP45N^Δ^C^Δ^‐GFP was predominately presented in nuclei, like that of RBP45‐GFP. However, the fluorescence of RBP45N‐GFP was similar to that of free GFP and evenly distributed throughout the cells. Thus, the central region (66–370 aa) of NtRBP45 might contain the putative NLS that direct nuclear localization of the protein.

### 3.3 Transient overexpression of NtRBP45 enhanced post‐transcriptional gene silencing

**FIGURE 3:**
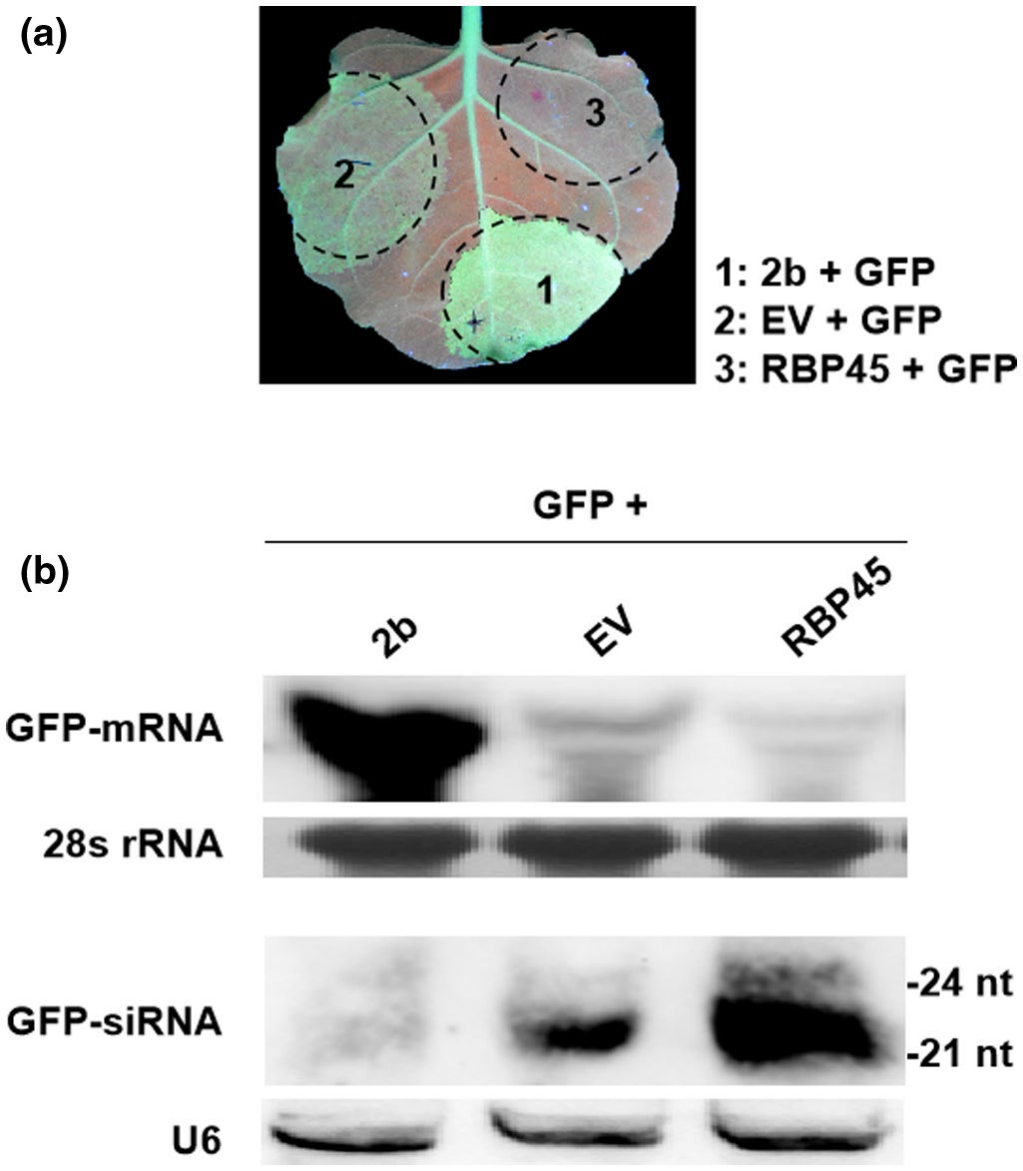
The tobacco NtRBP45 enhanced local GFP silencing in transgenic *Nicotiana benthamiana* line 16c. (A) The leaf infiltrated with *Agrobacterium* cells carrying 35S‐GFP plus empty vector (EV) pCAMBIA1305.1, pCAM‐RBP45, or pCAM‐TAV2b. Photographs were taken with long‐wavelength UV light at 3 days post‐infiltration. (B) Northern blot analysis of GFP‐mRNA and GFP‐siRNA accumulation in the infiltrated leaf areas shown in (A). Methylene blue stain of 28S rRNA was used as a loading control for GFP‐mRNA and the U6 snRNA hybridization served as a loading control for GFP‐siRNA

Given the up‐regulated transcription of *NtRBP45* upon TMV infection, we questioned if the protein was engaged in the antiviral defense mechanism, such as RNA silencing. A transient silencing suppression assay was then employed to test this hypothesis. The leaves of *N. benthamiana* line 16c that transgenically expresses GFP were infiltrated with a mixture of the *Agrobacterium* cells bearing pCAM‐RBP45 and pCAMBIA1300‐35S‐GFP. As controls, the *Agrobacterium* cells harboring either pCAM‐TAV2b that bears a known gene silencing suppressor TAV2b from *Tomato aspermy virus* (Li et al., 1999), or the empty vector pCAMBIA1305.1 were co‐infiltrated with *Agrobacterium* carrying pCAMBIA1300‐35S‐GFP. The green fluorescence was monitored at 3 dpi under UV light. As expected, the leaf patches receiving pCAM‐TAV2b plus pCAMBIA1300‐35S‐GFP showed bright green fluorescence due to the post‐transcriptional gene silencing (PTGS) suppressor activity of TAV2b, and the fluorescence in leaves receiving the empty vector and pCAMBIA1300‐35S‐GFP was significantly reduced but remained detectable (Figure 3a). Conversely, no green fluorescence but deep red was seen in the patches receiving pCAMBIA1300‐35S‐GFP and pCAM‐RBP45 (Figure 3a). In line with the visual observation, the GFP mRNA was hardly to be detected in the deep read patches receiving pCAMBIA1300‐35S‐GFP and pCAM‐RBP45 (Figure 3b). In contrast, GFP mRNA reached higher levels in mild green fluorescent patches receiving the empty vector and pCAMBIA1300‐35S‐GFP, and was the most abundant in the bright green fluorescent patches that had received pCAM‐TAV2b plus pCAMBIA1300‐35S‐GFP (Figure 3b). These suggested that transient expression of NtRBP45 greatly decreased the accumulation of the GFP mRNA, thereby resulting in GFP fluorescence quenching.

To test whether the fast declined GFP expression under NtRBP45 was correlated to RNA silencing, we analyzed the accumulation of GFP‐siRNAs that guide the specific decay of GFP mRNA at 3 dpi. Due to the presence of TAV2b, few 21 nt, and 24 nt, GFP‐siRNAs were detected in the patches receiving pCAMBIA1300‐35S‐GFP and pCAM‐TAV2b (Figure 3b). However, the amount of GFP‐siRNAs in the patches receiving pCAMBIA1300‐35S‐GFP and pCAM‐RBP45 was significantly higher than that in the patches receiving pCAMBIA1300‐35S‐GFP and the empty vector, indicating that NtRBP45 facilitated siRNA accumulation and thus enhanced a sense transgene‐induced PTGS locally.

**FIGURE 4:**
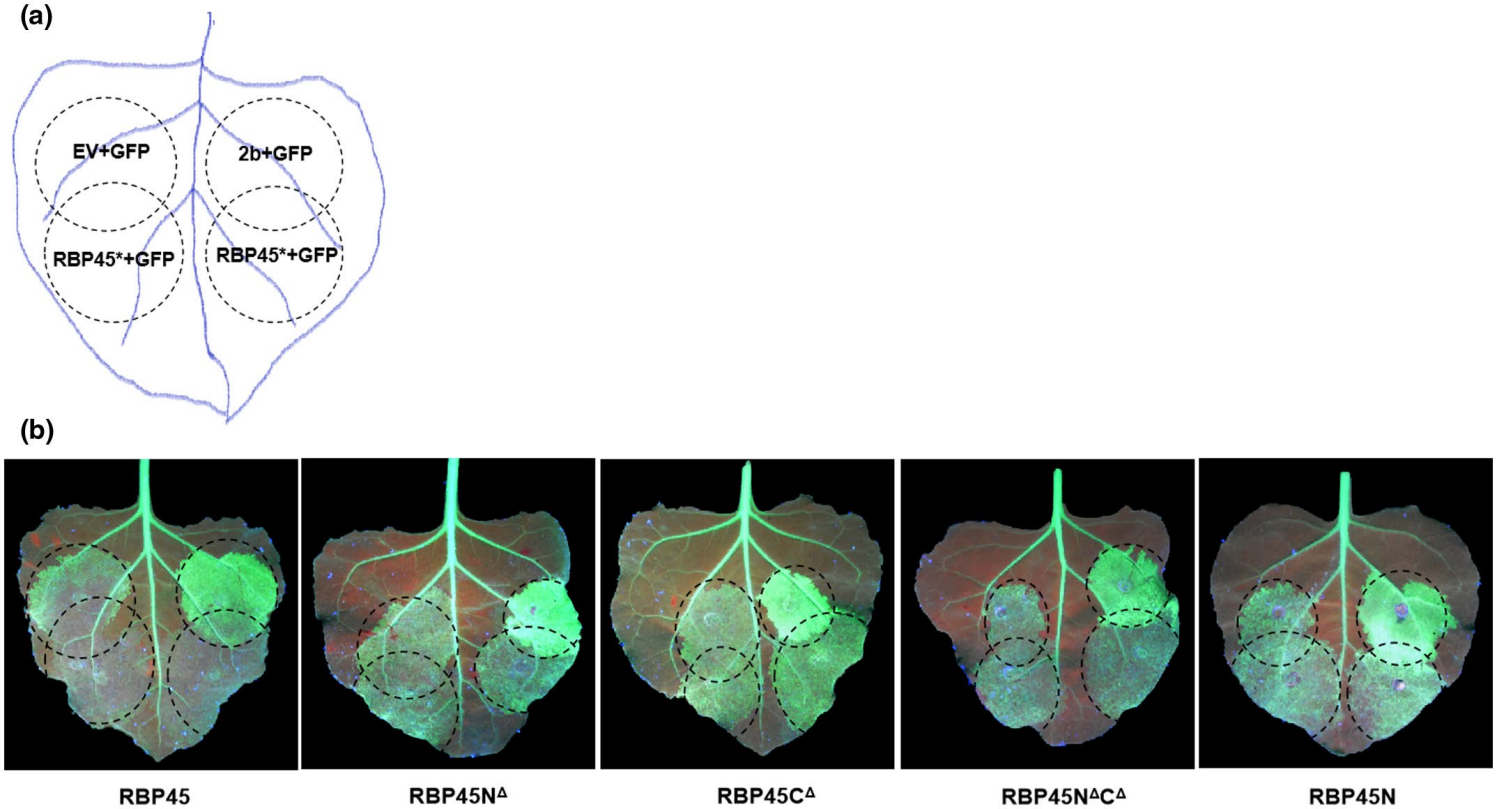
RNA silencing suppression activity of the RBP45 deletion mutants. (A) Patch design for overlapping co‐agroinfiltration in leaves of the transgenic N. benthamiana line 16c. RBP45* represents RBP45 and its deletion mutants RBP45N^Δ^, RBP45C^Δ^, RBP45N^Δ^C^Δ^, and RBP45N. (B) The representative leaves with overlapping co‐agroinfiltration. Photographs were taken at 3 days post‐infiltration with long‐wavelength UV light

The role of NtRBP45 in enhancing PTGS was further probed following a strategy of overlapping co‐infiltration depicted in Figure 4a. At 3 dpi, the green fluorescence exhibited at the overlapping areas was compared with that at the patches only receiving pCAMBIA1300‐35S‐GFP plus either pCAM‐TAV2b or the empty vector. As shown in Figure 4b, NtRBP45 greatly reduced the GFP fluorescence at the overlapping areas, suggesting that, as a PTGS enhancer, NtRBP45 partially restored gene silencing suppressed by TAV2b. With this strategy, we next tested the functions of the four NtRBP45 mutants (RBP45N^Δ^, RBP45C^Δ^, RBP45N^Δ^C^Δ^, and RBP45N) on RNA silencing. Visualization of the GFP expression under UV light showed that the green fluorescence at the patches receiving any of the deletion mutants plus pCAMBIA1300‐35S‐GFP was similar with that at the patches receiving the empty vector and pCAMBIA1300‐35S‐GFP (Figure 4b). Although three of four mutants (RBP45N^Δ^, RBP45C^Δ^, and RBP45N^Δ^C^Δ^) shared a similar subcellular localization with NtRBP45 (Figure 2), none of them was able to restore the TAV2b‐mediated gene silencing at the overlapping areas. Taken together, the data indicated that both N‐ and C‐termini were essential for NtRBP45 to enhance PTGS in *N. benthamiana*.

## 4 DISCUSSION

RBPs play roles in multiple biological mechanisms, including RNA silencing (Köster et al., 2017; Lorković, 2009). So far, a few RBPs have been shown to be involved in biogenesis of small no‐coding RNAs (sRNA), including siRNA and microRNA (miRNA) (Nussbacher & Yeo, 2018; Ren & Yu, 2012; Wu et al., 2013). Among them, the DCL family proteins are the most well‐known ones that act as the core silencing factors and are indispensable for either siRNA or miRNA biogenesis (Liu et al., 2009). In addition, HYPONASTIC LEAVES 1 (HYL1, also called DRB1), SERRATE (SE) and HIGH OSMOTIC STRESS GENE EXPRESSION 5 (HOS5) promote miRNA biogenesis (Chen et al., 2015; Dong et al., 2008; Ren & Yu, 2012), DRB4 and DRB7.2 are specifically engaged in siRNA biogenesis (Fukudome et al., 2011; Montavon et al., 2017), whereas DAWDLE (DDL), TOUGH (TGH) and MOS2 are critical in the biogenesis of both miRNAs and siRNAs (Ren et al., 2012; Wu et al., 2013; Yu et al., 2008; Zhang et al., 2018). Herein, transient overexpression of NtRBP45, a tobacco nuclear RBP, was demonstrated to facilitate GFP siRNA accumulation and enhance PTGS in *N. benthamiana*, indicating that the protein is an RNA silencing factor contributing to siRNA biogenesis. Notably, a recent study shows that AtRBP45b in *A. thaliana* can physically interact with CAP‐binding protein 20 (CBP20) (Muthuramalingam et al., 2017), a key component in producing miRNA (Kim et al., 2008). This report, combined with our current data, suggested that RBP45 might devote to not just siRNA accumulation but miRNA biogenesis.

The observation that the tobacco NtRBP45 facilitated GFP siRNA accumulation is reminiscent of a previous study wherein NpRBP45 of *N. plumbaginifolia* has been uncovered to specifically bind to oligouridylates (Lorković et al., 2000). Regarding an established role of 3'‐oligouridylates in stimulating the degradation of siRNA and miRNA (De Almeida et al., 2018), it could be inferred that the overexpressed NtRBP45 interacting with 3'‐oligouridylates might prevent RNA degradation, thus leading to the enhanced accumulation of GFP siRNAs that were detected in this study. Here, we also observed that the transcription of *NtRBP45* was significantly up‐regulated upon TMV infection, and in particular the transiently overexpressed NtRBP45 could partially restore the TAV2b‐mediated PTGS suppression in *N. benthamiana*. It is known that the viral RNA silencing suppressors counteract host RNAi‐based defense and thus promote virus infection (Wang et al., 2012; Wang & Metzlaff, 2005; Yang & Li, 2018). Taken together, our results emphasized the essential role of NtRBP45 in antiviral defense. In addition, since the *Cucumovirus* 2b protein targets multiple steps of RNA silencing through binding siRNA and AGOs, impairing 5' secondary siRNA genesis, or down‐regulation of RDR6 (Csorba et al., 2015), further elucidation of how NtRBP45 compromises the PTGS suppression capacity of TAV2b would be valuable for understanding its precise function in RNA silencing.

In conclusion, the study demonstrated that NtRBP45, a tobacco nuclear RBP, served as a PTGS enhancer and facilitated siRNA accumulation. The data collectively disclosed a novel RNA silencing factor that might play a fundamental role in siRNA‐based antiviral defense and merits further investigation.

## CONFLICT OF INTEREST

The authors declare no conflict of interest with the work described in the manuscript.

## AUTHOR CONTRIBUTIONS

W.Z., W.W., and W.L. designed the experiments, Z.Z., C.Z., P.D., and H.Y. carried out the experiments, W.Z., Z.Z., W.W., and W.L. analyzed the data, W.Z., W.W., and W.L. wrote the manuscript. All authors revised and approved the final version.

## FUNDING INFORMATION

This work was supported by the National Natural Science Foundation of China (31970126) and the Key Laboratory of Development and Application of Rural Renewable Energy, Ministry of Agriculture and Rural Affairs, China.

## Supporting information

Fig S1

Table S1
