# Supplementary material for: NtRBP45, a nuclear RNA‐binding protein of Nicotiana tabacum, facilitates post‐transcriptional gene silencing
Source: Plant Direct. 2020 Dec 24;4(12):e00294. doi: 10.1002/pld3.294 (PMC7880056; doi:10.1002/pld3.294)
Supplement: Supplementary file 1 — Fig S1 [file PLD3-4-e00294-s001.doc]

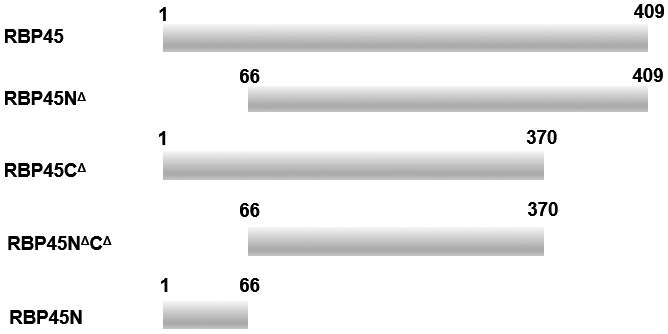


**Figure S1.** Schematic diagrams of NtRBP45 and its four deletion mutants RBP45N^Δ^, RBP45C^Δ^, RBP45N^Δ^C^Δ^ and RBP45N.
