# Supplementary material for: NtRBP45, a nuclear RNA‐binding protein of Nicotiana tabacum, facilitates post‐transcriptional gene silencing
Source: Plant Direct. 2020 Dec 24;4(12):e00294. doi: 10.1002/pld3.294 (PMC7880056; doi:10.1002/pld3.294)
Supplement: Supplementary file 2 — Table S1 [file PLD3-4-e00294-s002.doc]

Table S1. The primers used in this study

| **Application** | **Primer name** | **Nucleotide sequence (5'-3')^1^** |
| --- | --- | --- |
| **qPCR assay**  *NtRBP45*  *Ntactin*  **Constructs**  pRBP45  pRBP45-GFP | qPCR-RBP-F  qPCR-RBP-R  qPCR-actin-F  qPCR-actin-R  RBP45-F  RBP45-R  RBP45gfp-F | GGCTGCTGATGTGACGGATT  CCCACTGGTTTCTTGTTCGC  TTTCCGTTGTCCTGAGGTCC  TAGAGCCACCACTGAGCACA  ATGATGCCACAAAGTGGAGTTGC  TCACTGCTGTGGCTGCTGATAATT  GACGAGCTCGGGTACCATGATGCCACAAAGTGGA |
|  | RBP45gfp-R | TGGTGTCGACTCTAGACTGCTGTGGCTGCTGATA |
| pRBP45N^Δ^-GFP | N^△^gfp-F | GACGAGCTCGGGTACCATGCCGACGACAAACCCTAAC |
|  | RBP45gfp-R | ------ |
| pRBP45C^Δ^-GFP | RBP45gfp-F | ------ |
|  | C^△^gfp-R | TGGTGTCGACTCTAGACGCGCCACCACTACCACCCCA |
| pRBP45N^Δ^C^Δ^-GFP | N^△^gfp-F | ------ |
|  | C^△^gfp-R | ------ |
| pRBP45N-GFP | RBP45gfp-F | ------ |
|  | Ngfp-R | TGGTGTCGACTCTAGAAGCACCGTACTGTTGTTGC |
| pCAM-RBP45 | RBP-F | GGACTCTTGACCATGGATATGATGCCACAAAGTGGA |
|  | RBP-R | ATTCGAGCTGGTCACCTCACTGCTGTGGCTGCTGATA |
| pCAM-RBP45N^Δ^ | N^△^-F | GGACTCTTGACCATGGATATGCCGACGACAAACCCTAAC |
|  | RBP-R | ---- |
| pCAM-RBP45C^Δ^ | RBP-F | ---- |
|  | C^△^-R | GGACTCTTGAGGTCACCTCACGCGCCACCACTACCACCCCA |
| pCAM-RBP45N^Δ^C^Δ^ | N^△^-F | ---- |
|  | C^△^-R | ---- |
| pCAM-RBP45N | RBP-F | ---- |
| pCAM-TAV2b | 1. R   2b-F  2b-R | ATTCGAGCTGGTCACCTCAAGCACCGTACTGTTGTTGC  GGACTCTTGACCATGGATATGGCAAGCATCGAGATCCCT  ATTCGAGCTGGTCACCTCATTGATCGAGACACCAGTC |

1. The introduced restriction enzyme sites, including *Xba* I (TCTAGA), *Kpn* I (GGTACC), *Nco* I (CCATGG) and *Bst*E II (GGTCACC), were underlined.
